# Toxic Shock Syndrome in a 45-Year-Old Woman Possibly Associated with Tampon Use: A Case Report of Multiorgan Failure Due to *Streptococcus agalactiae*

**DOI:** 10.3390/diseases13110376

**Published:** 2025-11-16

**Authors:** Tina Zavidić, Ema Dejhalla, David Zahirović

**Affiliations:** 1 Istrian Health Centres, Lupoglav 10B, 52420 Lupoglav, Croatia; tina.zavidic@medri.uniri.hr; 2 Faculty of Medicine, University of Rijeka, Braće Branchetta 20, 51000 Rijeka, Croatia; david.zahirovic@medri.uniri.hr; 3 Family Medicine Office, Medical Centre for Occupational Health Rijeka, Verdieva 8, 51000 Rijeka, Croatia; 4 Community Health Centre of the Primorje-Gorski Kotar County, Krešimirova 52a, 51000 Rijeka, Croatia

**Keywords:** menstruation, sepsis, shock, tampon

## Abstract

Background: Toxic shock syndrome (TSS) is a rare but potentially fatal condition most often caused by *Staphylococcus aureus* or *Streptococcus pyogenes*. However, other streptococcal species, including *Streptococcus agalactiae (group B Streptococcus (GBS))*, can also cause TSS, sometimes leading to severe complications, such as multiorgan failure. Case Description: We report the case of a 45-year-old woman who developed TSS associated with tampon use. She presented with fever, chills, hypotension, and leg pain, progressing rapidly to septic shock and multiorgan failure. Blood and urine cultures revealed *S. agalactiae group B*, while a gynecological examination identified *Ureaplasma urealyticum* and *S. agalactiae*. Imaging demonstrated bilateral pneumonic infiltrates and pleural effusion. The patient required intensive care, vasopressor support, and broad-spectrum antibiotic therapy, leading to full clinical recovery. Discussion: Despite advances in tampon design, menstrual TSS remains a significant clinical concern. Early symptoms may be nonspecific, but rapid progression highlights the need for timely recognition and intervention. Although *S. agalactiae* is an uncommon cause of TSS, it should be considered in relevant clinical scenarios. Prompt empirical antibiotic therapy, followed by targeted treatment based on culture results, along with supportive intensive care, is essential to improve outcomes. Conclusions: Menstrual TSS continues to pose a serious health risk. Physicians should maintain a high index of suspicion in tampon users presenting with fever, rash, and shock. Early diagnosis and rapid initiation of appropriate therapy are crucial to reducing morbidity and mortality.

## 1. Introduction

Toxic shock syndrome (TSS) is a rare, life-threatening, toxin-mediated disease characterized by a constellation of clinical features, including high fever, hypotension, a diffuse erythematous rash, and involvement of multiple organ systems. The syndrome is most associated with toxigenic strains of Staphylococcus aureus or *group A Streptococcus (Streptococcus pyogenes)* [1]. Certain other streptococcal strains can also contribute to the development of TSS. The condition most frequently occurs with the use of menstrual tampons. The fatality rate for streptococcal TSS can exceed 50%, whereas the mortality rate for non-streptococcal TSS is below 3% [2].

Since its initial description in the late 1970s and early 1980s, TSS has remained a significant clinical and public health concern due to its rapid onset, high morbidity, and potential mortality [3]. The disease represents a classic example of how bacterial exotoxins, specifically superantigens, can dysregulate the immune system, triggering an overwhelming systemic inflammatory response. In the case of *S. aureus*, the toxins toxic shock syndrome toxin-1 (TSST-1) and staphylococcal enterotoxins play central roles, while in *S. pyogenes* infections, streptococcal pyrogenic exotoxins (SPEs) such as SpeA and SpeC are key mediators. These superantigens bypass conventional antigen processing by binding directly to major histocompatibility complex (MHC) class II molecules and T-cell receptors, resulting in polyclonal T-cell activation and the release of massive amounts of cytokines, including interleukin (IL)-1, IL-2, tumor necrosis factor-alpha (TNF-α), and interferon-gamma (IFN-γ). The downstream effect is a cytokine storm that leads to vascular leakage, hypotension, multiorgan dysfunction, and shock [4].

Although menstrual-related TSS has become less frequent due to changes in tampon composition, absorbency standards, and public health campaigns promoting safer usage practices, cases continue to occur. Non-menstrual TSS, often associated with surgical wounds, soft tissue infections, burns, or postpartum infections, is now proportionally more common than the menstrual form. Nevertheless, tampon-associated TSS continues to present a unique diagnostic and therapeutic challenge, particularly when caused by less common organisms, such as Group B Streptococcus (GBS) [5].

*GBS* is a Gram-positive β-hemolytic bacterium most widely recognized as a leading cause of neonatal sepsis, meningitis, and pneumonia. However, it is also an important cause of invasive disease in adults, particularly in the elderly, immunocompromised individuals, and pregnant women [6]. *GBS* colonizes the gastrointestinal and genitourinary tracts in up to 30% of healthy women and can ascend from the vaginal mucosa to cause bacteremia, endometritis, and urinary tract infections. While its role in TSS is exceedingly rare, documented cases demonstrate that *GBS* can produce exotoxins with superantigenic activity, leading to a clinical picture indistinguishable from classic TSS [7].

The association between tampon use and TSS was first recognized in the early 1980s following a surge in cases linked to highly absorbent synthetic tampons. It is hypothesized that prolonged tampon retention alters the vaginal microenvironment, increasing oxygen availability and pH, conditions that favor the proliferation of toxigenic Staphylococcus and Streptococcus species. Additionally, microabrasions caused by tampon insertion or removal may disrupt mucosal barriers, facilitating bacterial translocation into the systemic circulation. Despite improvements in tampon design and increased public awareness, these risk factors persist, particularly when tampons are used for extended periods or when individuals are colonized with toxigenic bacterial strains [8].

The clinical course of TSS is often fulminant, with rapid progression from initial nonspecific symptoms to severe systemic illness. Early manifestations may include fever, myalgias, gastrointestinal disturbances, and malaise, symptoms that are easily mistaken for common viral infections. As the disease evolves, patients typically develop hypotension, a characteristic sunburn-like rash, and signs of multiorgan involvement, such as acute kidney injury, hepatic dysfunction, coagulopathy, and respiratory failure. The high mortality associated with streptococcal TSS underscores the importance of early recognition and aggressive management, including prompt initiation of antimicrobial therapy, hemodynamic support, and source control [9].

In this case report, we present a rare occurrence of TSS caused by *GBS* in a 45-year-old woman associated with tampon use. The patient’s clinical course was complicated by septic shock and multiorgan failure, requiring intensive care support. This case underscores the importance of considering *GBS* as a potential etiological agent in menstrual-associated TSS and highlights the critical role of early diagnosis and targeted therapy in improving outcomes. By examining this case in the context of the existing literature, we aim to contribute to the growing understanding of atypical presentations of TSS and emphasize the continued relevance of this syndrome in modern clinical practice [10].

## 2. Case Description

A 45-year-old female patient presented to a family medicine office due to a fever reaching 39.6 °C. She had previously vomited and had two loose stools. She reported that, during the night, she was woken up by chills and shaking, and her temperature was 37.6 °C. Her condition improved after taking antipyretics, but her temperature rose to 38.8 °C in the morning, accompanied by general malaise and muscle pain in her legs. She complained of a subjective feeling of being unable to take a full breath. Upon examination, she was oriented, severely weak, mildly tachypneic (24/min), hemodynamically unstable with a blood pressure of 90/55 mmHg, and tachycardic. The oxygen saturation level was 97%. Meningeal signs were negative. A mild erythema and pinpoint rash that blanched under pressure were observed on the skin of the torso and lower extremities. The heart had a regular rhythm, with clear tones. The bilateral lung sounds were normal. The pharynx showed no exudate, and the tongue was coated. The lymph nodes were not palpable. The abdomen was soft and elastic, with mild tenderness in the epigastric area. Peristalsis was audible. Lumbar percussion was negative. The extremities were symmetrical, without edema. She was referred to emergency care due to suspicion of sepsis development.

The patient’s initial presentation was non-specific and could have been mistaken for a viral syndrome, gastrointestinal infection, or influenza-like illness. However, several clinical features raised immediate concern for a severe systemic process. The combination of high-grade fever, rigors, tachycardia, hypotension, and a blanching rash was a classic warning sign indicating toxin-mediated disease or sepsis. Additionally, the subjective dyspnea, despite normal oxygen saturation, hinted at systemic inflammatory involvement rather than primary respiratory pathology. Early recognition of this pattern is essential, as prompt referral and escalation of care can significantly reduce the morbidity and mortality associated with TSS.

In March 2024, approximately nine months before the onset of the present illness, the patient underwent a total thyroidectomy due to papillary thyroid carcinoma, and ablation with iodine-131 was performed. She takes 125 μg of levothyroxine.

Her past medical history was significant for a recently treated papillary thyroid carcinoma, including total thyroidectomy and radioiodine ablation, which may have contributed to subtle alterations in her immune response. Although the patient had no autoimmune disease, chronic immunosuppression, or diabetes, metabolic changes following thyroidectomy might have subtly affected immune function. Her only chronic medication was levothyroxine, and she reported strict adherence to therapy.

Upon admission to emergency care, laboratory tests, blood and urine cultures, and a pulmonary CT angiography due to suspicion of pulmonary embolism were performed. The laboratory results showed the following values: platelets 60 × 10⁹/L, hemoglobin 110 g/L, aspartate aminotransferase (AST) 61 U/L, ALT 58 U/L, alanine aminotransferase (ALP) 364 U/L, gamma-glutamyl transferase (GGT) 275 U/L, C-reactive protein (CRP) 194.2 mg/L, procalcitonin (PCT) 1.07 ng/mL, leukocytes 9 × 10⁹/L, neutrophils 70.9%, eGFR 90 mL/min/1.73 m^2^, and lactate dehydrogenase (LD) 281 U/L (Table 1).

The initial laboratory investigations revealed thrombocytopenia, elevated inflammatory markers (CRP and procalcitonin), and evidence of hepatic involvement, with elevated levels of transaminases, alkaline phosphatase, and gamma-glutamyl transferase. These findings were consistent with a systemic inflammatory response syndrome (SIRS) complicated by early multiorgan dysfunction. The normal leukocyte count with neutrophilic predominance suggested a bacterial etiology but did not exclude severe sepsis, as leukopenia or leukocytosis may not always be present in TSS. The marked elevation of CRP and procalcitonin levels strongly supported a bacterial cause rather than a viral or autoimmune process.

*GBS* was isolated from blood and urine cultures, prompting a gynecological consultation. Microbiological investigations were performed using automated systems (BacT/ALERT and VITEK 2). *S. agalactiae* group B was identified using MALDI-TOF mass spectrometry. Antibiotic susceptibility testing revealed sensitivity to penicillin, ampicillin, and clindamycin, and resistance to tetracycline. These findings guided the use of targeted antimicrobial therapy with ampicillin and clindamycin. Clindamycin plays a key role in TSS management because it inhibits bacterial toxin production even during stationary-phase growth. Its combination with β-lactam antibiotics provides both bactericidal activity and toxin suppression, which is critical for toxin-mediated syndromes [11]. In our case, this combination therapy, along with early hemodynamic support and close monitoring, led to full recovery within 10 days. Antimicrobial susceptibility testing was performed by the disk diffusion method and interpreted according to the European Committee on Antimicrobial Susceptibility Testing (EUCAST) clinical breakpoints, version 14.0, 2024 [12]. Her last menstruation began five days prior, with otherwise regular cycles lasting for 4–5 days. She occasionally used tampons, changing them regularly. She regularly underwent gynecological checkups, with no previous issues, and she had two uneventful vaginal deliveries. The gynecological exam revealed a cervical polyp protruding from the cervical os, measuring 1.5 cm. An ultrasound examination showed a hyperechoic endometrium measuring 4.5 mm and no free fluid in Douglas’ pouch, and other findings were normal. Cervical swabs were taken, and *Ureaplasma urealyticum* and *GBS* were isolated.

The isolation of *GBS* from both blood and urine cultures was a pivotal finding that guided subsequent management decisions. 

The identification of a cervical polyp was another noteworthy finding. While benign cervical polyps are common and usually asymptomatic, they can serve as potential foci for bacterial colonization and biofilm formation, increasing the risk of ascending infection. The lack of free fluid in the Douglas pouch indicated no pelvic abscess or overt peritoneal involvement at the time of imaging.

**Figure 1:**
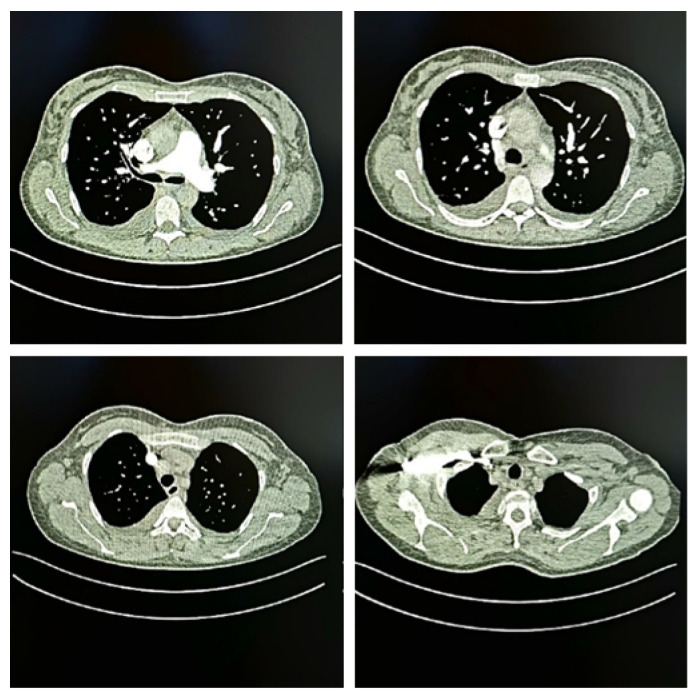
CT pulmonary angiography of the patient presented in this case, showing bilateral pneumonic infiltrates and pleural effusion.

The CT pulmonary angiography indicated bilateral pneumonic infiltrates and pleural effusion (Figure 1). The imaging findings of bilateral pneumonic infiltrates and pleural effusion reflected systemic dissemination of the infection and possible development of acute respiratory distress syndrome (ARDS), a hallmark of severe TSS. Pleural effusion in this context is typically non-infectious and results from increased vascular permeability secondary to cytokine-mediated capillary leakage rather than direct pulmonary infection. These findings corroborated the clinical impression of a severe systemic inflammatory response with pulmonary involvement.

**Figure 2:**
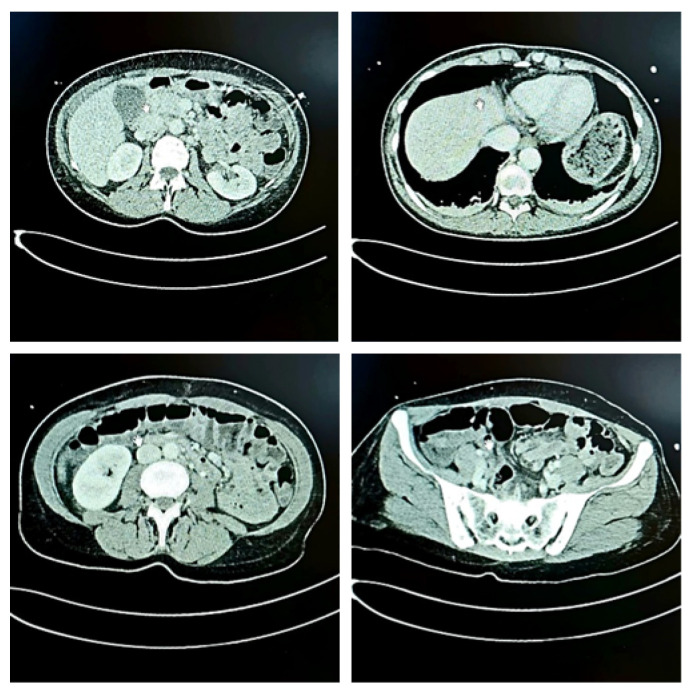
CT scan of the abdomen and pelvis of the patient presented in this case, showing mild free fluid in the posterior fornix and endometrial imbibition.

The CT scan of the abdomen and pelvis showed a small amount of free fluid with marginal imbibition in the area of the posterior fornix of the vagina on the left, with slightly more intense imbibition of the endometrium and some liquid content (Figure 2).

The abdominal and pelvic imaging further supported the diagnosis of a urogenital source of sepsis. The small volume of free fluid and endometrial changes were likely inflammatory rather than infectious in nature, reflecting the mucosal immune response to ascending bacterial invasion. These findings, in combination with the microbiological results, provided a comprehensive picture of TSS originating in the genitourinary tract.

The patient was diagnosed with sepsis caused by *S. agalactiae* originating in the urogenital system and septic shock with multiorgan failure and was kept for treatment in the intensive care unit. She was febrile up to 39.4 °C during the first three days, then subfebrile up to 37.6 °C for two days, and afebrile for the rest of the stay. She required low-dose norepinephrine support and then remained hemodynamically stable, maintaining adequate oxygenation with room air. She was treated antimicrobially with ampicillin and clindamycin, along with a probiotic. She received intravenous electrolyte solutions, diuretics, gastroprotection, thromboprophylaxis, antipyretics, and her chronic therapy. She was discharged on the tenth day with continued treatment with 3 × 1 g of amoxicillin and 500 mg of azithromycin for another five days. The decision to continue oral amoxicillin with azithromycin after discharge was precautionary, reflecting empirical coverage for possible residual genitourinary or respiratory colonization, given the initial detection of *Ureaplasma urealyticum*. No microbiological evidence of coinfection was confirmed. The laboratory parameters were satisfactory at the check-up after 10 days, and she was left to further checks by the family doctor, which were also satisfactory.

The patient’s clinical course was typical for severe TSS, with persistent high-grade fever during the initial phase of illness, followed by a gradual defervescence as systemic inflammation subsided. The need for vasopressor support highlighted the severity of circulatory collapse, a defining feature of TSS. The decision to use norepinephrine as the first-line vasopressor was consistent with international sepsis guidelines, given its efficacy in restoring perfusion pressure with minimal arrhythmogenic risk. The combination of ampicillin and clindamycin provided synergistic coverage—with ampicillin targeting cell wall synthesis as a β-lactam antibiotic and clindamycin suppressing toxin production as a protein synthesis inhibitor—a critical factor in toxin-mediated diseases such as TSS.

Supportive care measures, including fluid resuscitation, electrolyte management, and thromboprophylaxis, were essential components of the patient’s treatment. The addition of a probiotic aimed to mitigate antibiotic-associated dysbiosis, which can be particularly pronounced in patients receiving broad-spectrum antibiotics. The patient’s favorable response, characterized by rapid hemodynamic stabilization and resolution of systemic inflammation, reflected the effectiveness of timely, targeted therapy.

The patient achieved full clinical recovery by day 10 of hospitalization. At discharge, inflammatory markers normalized (with CRP < 5 mg/L, PCT < 0.05 ng/mL, a platelet count of 250 × 10⁹/L, and AST/ALT within a normal range). At the 4-week follow-up, she remained asymptomatic, with no organ dysfunction or laboratory abnormalities. This outcome underscores the importance of early diagnosis, aggressive supportive care, and pathogen-directed antimicrobial therapy in improving survival and functional outcomes in TSS patients.

## 3. Discussion

Toxic shock syndrome (TSS) is a rare but severe toxin-mediated disease most commonly caused by *Staphylococcus aureus* or *Streptococcus pyogenes*, although other streptococcal species may also contribute [13]. While TSS is typically associated with menstrual tampon use, non-menstrual forms related to surgical wounds or soft-tissue infections are also described. Our case presents an uncommon occurrence of TSS in a 45-year-old woman, adding to the limited number of reported adult cases.

### 3.1. Case-Based Analysis and Literature Context

The patient presented with fever, hypotension, a blanching rash, and evidence of multiorgan involvement, features fulfilling the CDC clinical criteria for TSS [14,15]. The temporal association between the onset of symptoms and menstruation, combined with tampon use, initially suggested menstrual TSS. However, a definitive causal link cannot be established; therefore, this case should be considered as possibly associated with tampon use rather than directly caused by it. Similar uncertainty has been noted in other reported *GBS*-related TSS cases, where genitourinary colonization or gynecological factors, rather than tampon use alone, likely served as the infectious source [16,17,18].

Blood, urine, and cervical cultures all yielded *S. agalactiae*, confirming bacteremia and a genitourinary origin. The detection of *Ureaplasma urealyticum* was likely incidental, though a polymicrobial environment could have enhanced toxin production. The presence of a cervical polyp, as found in our patient, may have provided a site for bacterial adherence and biofilm formation, facilitating systemic dissemination. These findings emphasize that even in the absence of improper tampon use, menstrual-related TSS can occur when host or local factors permit bacterial invasion. This case is distinctive for several reasons. First, it illustrates *S. agalactiae* as an uncommon etiologic agent of menstrual-associated TSS. Second, the presence of a cervical polyp may have facilitated bacterial colonization, while the history of recent thyroidectomy could represent a subtle host factor affecting immune response. Together, these findings highlight the need for heightened clinical awareness of atypical streptococcal causes of TSS and the importance of thorough microbiological evaluation in menstruating women presenting with septic shock.

Our patient’s clinical evolution—rapid onset of sepsis, multiorgan dysfunction, and full recovery after targeted therapy—mirrors outcomes reported in other *GBS*-TSS cases. According to previously published data, *GBS*-related TSS has been documented in fewer than 20 adult cases, predominantly in women of reproductive age, with mortality rates between 15% and 30% [19,20,21,22].

### 3.2. Pathophysiological Considerations

The pathogenesis of TSS revolves around superantigen-mediated immune activation. Superantigens bypass normal antigen presentation by linking MHC class II molecules with T-cell receptors, causing massive cytokine release (IL-1, IL-2, TNF-α, IL-6, and IFN-γ), endothelial dysfunction, and multiorgan injury [23]. While this mechanism is well established for *S. aureus* and *S. pyogenes*, emerging evidence suggests that certain strains of *GBS* may also produce exotoxins with superantigen-like activity capable of triggering a comparable cytokine storm. Although superantigen gene profiling or toxin assays were not performed in our case, this limitation has been acknowledged. Taken together, these findings indicate that *GBS* may, under certain conditions, induce a toxic shock-like syndrome via mechanisms analogous to classical superantigen activation, though this remains to be confirmed experimentally [24].

### 3.3. Differential Diagnosis and Clinical Implications

The initial presentation of TSS is often nonspecific and may mimic viral infections, scarlet fever, or drug reactions [24]. In this case, the combination of rash, hypotension, and elevated inflammatory markers quickly oriented clinicians toward a toxin-mediated process. For practical purposes, clinicians should obtain blood, urine, and vaginal cultures in any menstruating woman presenting with fever and shock and consider non-group A streptococci as potential causes.

Empirical antibiotic therapy should include clindamycin plus a β-lactam active against streptococci and staphylococci and should be refined once susceptibility data are available. Adjunctive therapies, including fluid resuscitation, vasopressors, and consideration of intravenous immunoglobulin (IVIG), may improve outcomes in severe cases [25].

### 3.4. Comparison with Previous Reports

The rarity of *S. agalactiae*-associated TSS means that each well-documented case adds important insights.

Recent large-scale work by Tsuchihashi et al., based on a nationwide Japanese cohort of 301 adult patients with streptococcal toxic shock syndrome caused by β-hemolytic streptococci (*S. pyogenes, S. agalactiae*, and *S. dysgalactiae subsp. equisimilis*), showed that *S. agalactiae* accounted for 17.6% of cases, with mortality rates approaching 50–60%. This finding highlights that *GBS*-related TSS is not a benign variant but a life-threatening infection with outcomes comparable to group A streptococcal TSS [26].

Similarly, Inada et al., in a single-center retrospective study, compared the clinical characteristics of STSS due to different β-hemolytic streptococci and found that *S. agalactiae* infections were more likely to originate from the genitourinary tract and to affect older adults, yet could still cause fulminant multiorgan failure [27].

Kawai et al. reported a fatal non-pregnant adult case of *S. agalactiae* TSS-like syndrome, where genomic analysis identified virulence gene clusters linked to enhanced invasiveness and cytokine activation, suggesting that certain *GBS* strains possess toxin-mediated pathogenic potential similar to *S. pyogenes* [28].

Finally, Yun and Johnson described recurrent TSS episodes caused by *S. dysgalactiae* and, later, by *GBS*, demonstrating that host susceptibility and possible immune priming may contribute to recurrence [29].

Together, these studies confirm that *S. agalactiae* is a bona fide cause of TSS in adults and that its incidence may be underestimated due to diagnostic bias toward *S. aureus* and GAS. 

Our patient adds to this literature by illustrating a nonfatal case with a genitourinary focus, tampon use, and rapid recovery following early recognition and targeted therapy, showing that prompt management can markedly improve outcomes in *GBS*-associated TSS.

### 3.5. Summary

This case underscores the importance of considering *S. agalactiae* as a potential, though rare, cause of TSS. It also highlights the value of early recognition, broad initial antimicrobial coverage, and subsequent microbiological confirmation. While tampon use was temporally related, causality cannot be assumed. The case should therefore be interpreted as possibly associated with tampon use, contributing to the understanding of atypical presentations of TSS in women of reproductive age.

## 4. Conclusions

Toxic shock syndrome remains a rare but life-threatening condition that requires immediate recognition and intervention. This case illustrates that *GBS*, although less common than *S. aureus* or *S. pyogenes*, can cause severe TSS associated with tampon use and result in rapid clinical deterioration. Healthcare providers should consider TSS in the differential diagnosis of tampon users presenting with fever, hypotension, rash, and signs of organ dysfunction. Early identification of the causative organism, prompt initiation of appropriate antimicrobial therapy, and intensive supportive care are crucial for improving patient outcomes and reducing mortality.

## Figures and Tables

**Table 1:** Key clinical and laboratory data of the patient during critical illness (day 1 of hospitalization).

Parameter	Value	Reference Range	Interpretation
Temperature	39.6 °C	—	High fever
Blood pressure	90/55 mmHg	>100/60 mmHg	Hypotension
Heart rate	110 bpm	60–100 bpm	Tachycardia
Platelets	60 × 10⁹/L	150–400 × 10⁹/L	Thrombocytopenia
CRP	194.2 mg/L	<5 mg/L	Markedly elevated
PCT	1.07 ng/mL	<0.05 ng/mL	Elevated
AST/ALT	61/58 U/L	<40 U/L	Mild hepatic dysfunction
ALP/GGT	364/275 U/L	<120/<40 U/L	Cholestatic elevation
eGFR	90 mL/min	>90 mL/min	Normal renal function

## Data Availability

Dataset available on request from the authors.
